# Au Nanoflowers for Catalyzing and In Situ Surface-Enhanced Raman Spectroscopy Monitoring of the Dimerization of *p*-Aminothiophenol

**DOI:** 10.1021/acsomega.1c03933

**Published:** 2021-09-21

**Authors:** Jingwen Ba, Yandong Han, Xiaoyu Zhang, Lijuan Zhang, Shuhan Hui, Zhenzhen Huang, Wensheng Yang

**Affiliations:** † State Key Laboratory of Inorganic Synthesis and Preparative Chemistry, College of Chemistry, Jilin University, Changchun 130012, China; ‡ Institute of Molecular Plus, Tianjin University, Tianjin 300072, China

## Abstract

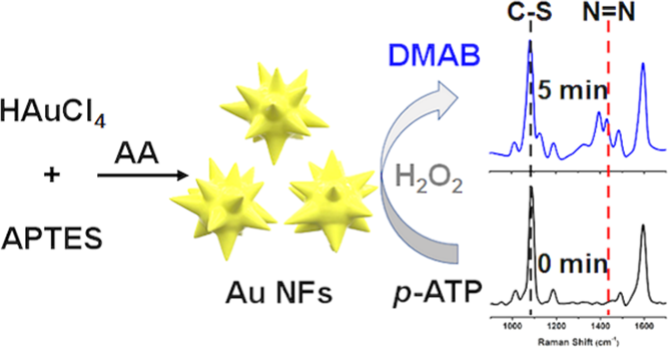

In this work, we demonstrated a facile approach for fabrication of Au nanoflowers (Au NFs) using an amino-containing organosilane, 3-aminopropyltriethoxysilane (APTES), as a shape-directing agent. In this approach, the morphology of the Au particles evolved from sphere-like to flower-like with increasing the concentration of APTES, accompanied by a red shift in the localized surface plasmon resonance peak from 520 to 685 nm. It was identified that the addition of APTES is profitable to direct the preferential growth of the (111) plane of face-centered cubic gold and promote the formation of anisotropic Au NFs. The as-prepared Au NFs, with APTES on their surface, presented effective catalytic and surface-enhanced Raman scattering (SERS) performances, as evidenced by their applications in catalyzing the dimerization of *p*-aminothiophenol and monitoring the reaction process via in situ SERS analysis.

## Introduction

Au nanoflowers (Au NFs) have aroused particular interests owing to the existence of “hot spots” distributed on their rough surface and thus a significantly enhanced electromagnetic field around the junctions and sharp tips, beneficial for their applications in catalysis and surface-enhanced Raman scattering (SERS).^1−12^ Usually, surfactants are necessary to be employed as shape-directing agents to direct the preferential growth of the Au particles, along a definite lattice plane of face-centered cubic (fcc) gold, to form the anisotropic Au NFs.^13−20^ For example, Wang et al. reported the growth of anisotropic Au NFs by passivating the (111) facet of fcc gold using (1-hexadecyl)trimethylammonium chloride as a “face-blocking” agent.^21^ Hwang et al. reported the fabrication of Au NFs with broad NIR adsorption by directing the preferential growth along the (111) plane of fcc gold using a gemini cationic surfactant, *N*,*N*,*N*′*N*′-tetramethyl-*N*,*N*′-ditetradecylethane-1,2-diaminium bromide.^22^ Sadik et al. demonstrated the synthesis of Au NFs with tunable surface roughness using pyromellitic dianhydride-*p*-phenylene diamine as both shape-directing and reducing agents.^23^ Kim et al. demonstrated a covalently capped seed-mediated growth approach for the synthesis of flower-like Au nanostructures by employing 6-mercaptohexanol and cetyltrimethylammonium bromide as shape-directing agents.^24^

3-Aminopropyltriethoxysilane (APTES) is an amino-containing organosilane, which is commonly used for the modification of the silica surface and can provide active amino sites for anchoring or growth of Au particles.^25−27^ In this work, we demonstrated a facile approach for the synthesis of Au NFs using APTES as a shape-directing agent. In this approach, the morphology of the Au particles evolved from sphere-like to flower-like with increasing the concentration of APTES introduced into the reaction solutions, accompanied with a red shift of the localized surface plasmon resonance (LSPR) peak (λ_max_) from 520 to 685 nm, attributed to the promoted preferential growth of the (111) plane of fcc gold by APTES. The resulting Au NFs presented effective catalytic and SERS performances,^28^ making them act as both the catalyst to promote the dimerization of *p*-aminothiophenol and as an in situ SERS substrate to monitor the reaction process.

## Results and Discussion

**Figure 1:**
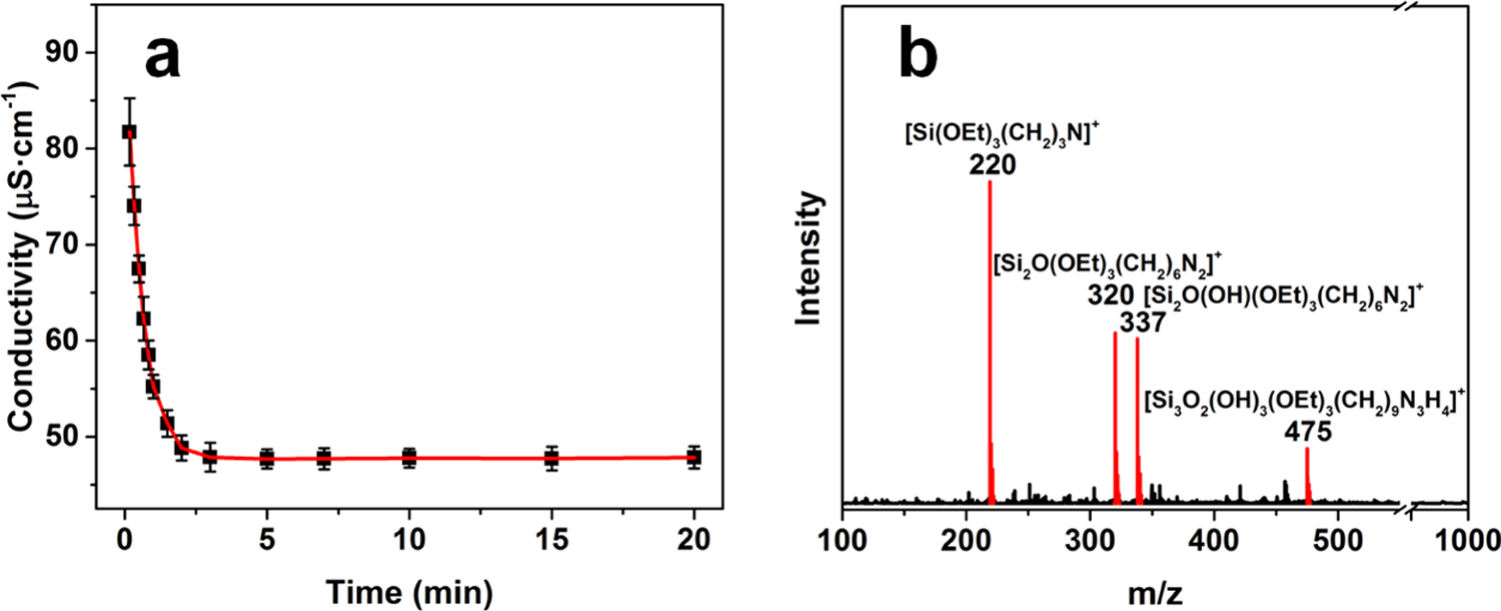
(a) Temporal evolution in the conductivity of the aqueous solution of APTES (3 mM). (b) Mass spectrum of the 3 mM APTES solution taken out at 5 min.

**Scheme 1:**
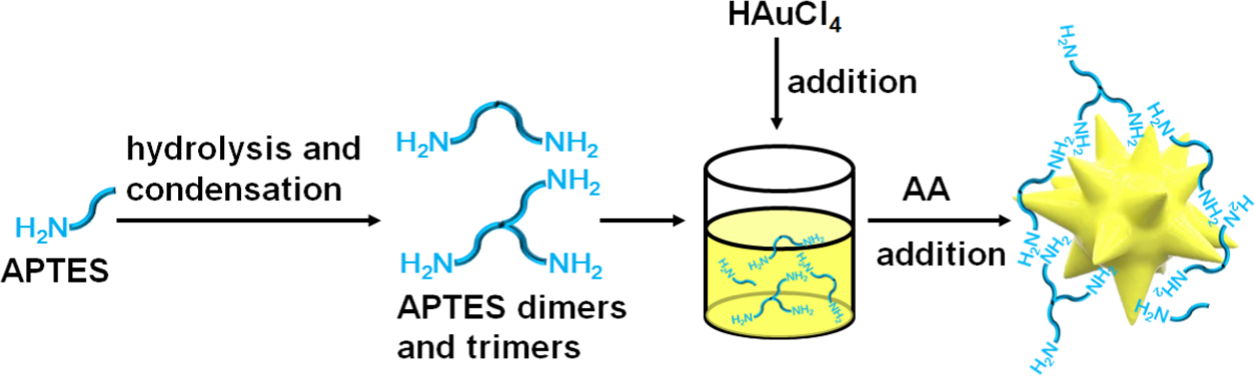
Schematic Illustration of the Synthesis Process of the Au NFs

In this work, an amino-containing organosilane agent, APTES, was used to direct the growth of Au NFs. In this approach, first, APTES was added in water to undergo self-catalyzed hydrolysis and condensation^29,30^ to form oligomers, such as dimers and trimers. Then, HAuCl_4_ was added in the APTES solution, followed by the addition of a reductant (AA) to form the Au NFs (Scheme 1). In our experiments, first conductivity measurements were carried out to evaluate the hydrolysis and condensation of APTES in an aqueous solution.^31^ An aqueous solution of APTES (3 mM) presented a conductivity of 82 μS·cm^–1^ after 10 s of the reaction, which dropped sharply to 48 μS·cm^–1^ and then remained less changed after 3 min of the reaction (Figure 1a), suggesting the much fast hydrolysis over the condensation of APTES at the initial stage of the reaction (0–3 min) and the well-balanced hydrolysis and condensation at the late stage of the reaction (3–20 min).^32^ According to the negative differentiation curve of the conductivity (Figure S1), the maximum condensation rate of APTES was derived to be ≥46 μS·cm^–1^·min^–1^.^33^ The mass spectrum of the solution, taken out after 5 min upon the addition of APTES, presented a peak corresponding to unhydrolyzed APTES, [Si(OEt)_3_(CH_2_)_3_N]^+^ (*m*/*z* = 220), as well as peaks corresponding to a dimeric product with one silanol group, [Si_2_O(OH)(OEt)_3_(CH_2_)_6_N_2_]^+^ (*m*/*z* = 337) or without a silanol group, [Si_2_O(OEt)_3_(CH_2_)_6_N_2_]^+^ (*m*/*z* = 320). In addition, a peak corresponding to a trimeric product with three silanol groups, [Si_3_O_2_(OH)_3_(OEt)_3_(CH_2_)_9_N_3_H_4_]^+^ (*m*/*z* = 475), was also observed in the spectrum (Figure 1b). These results indicated that APTES undergoes hydrolysis and the hydrolyzed APTES condenses into dimers and trimers in the aqueous solution, catalyzed by its own amino group.^34^ Therefore, in our experiments, the aqueous solution of APTES was kept at room temperature for 5 min, allowing the hydrolysis and condensation of APTES to reach the balance, before mixing it with the aqueous solution of HAuCl_4_.

**Figure 2:**
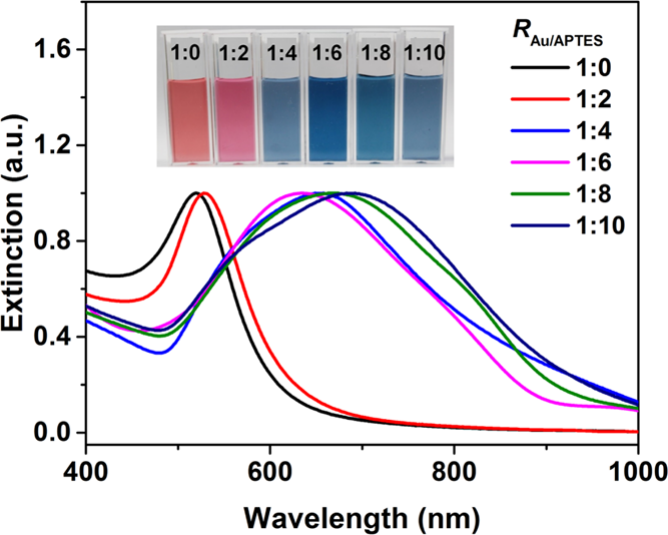
Normalized UV–vis spectra of the Au hydrosols prepared at different molar ratios of HAuCl_4_ and APTES (*R*_Au/APTES_) from 1:0 to 1:10. The inset shows photographs of the corresponding hydrosols.

**Figure 3:**
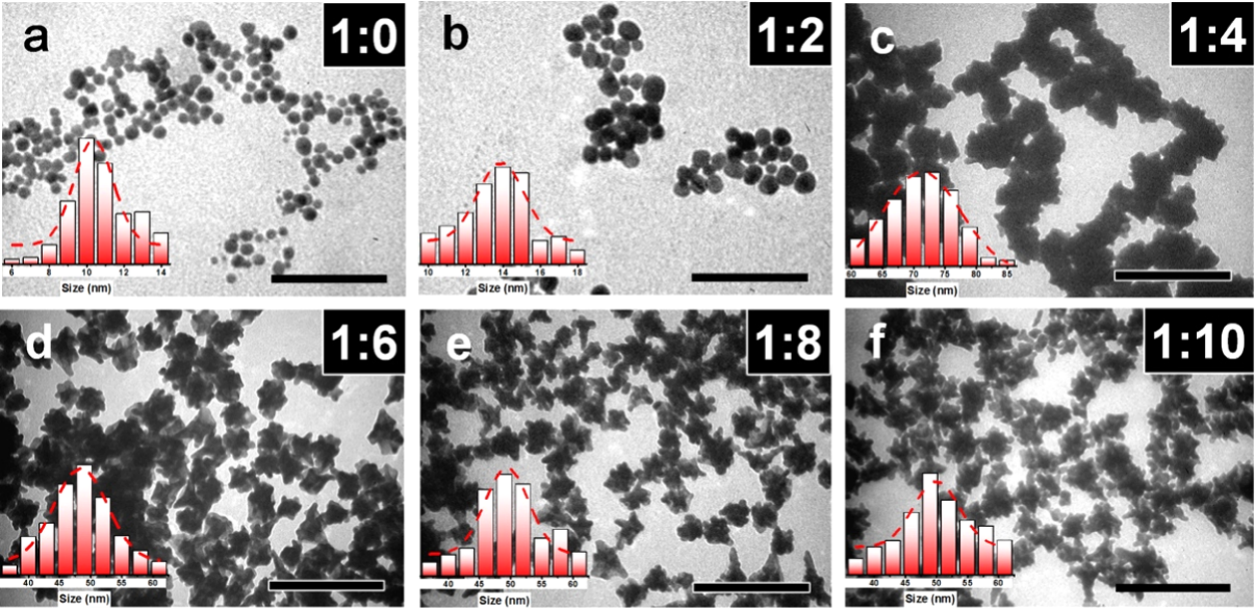
TEM images of the Au particles prepared at *R*_Au/APTES_ of (a) 1:0, (b) 1:2, (c) 1:4, (d) 1:6, (e) 1:8, and (f) 1:10. The inset gives histograms of the numerical size distribution of the particles. The scale bars in TEM images are 100 nm in (a, b) and 200 nm in (c–f).

In the subsequent procedure, the aqueous solution of HAuCl_4_ (100 mM) was mixed with different volumes of the APTES solution (3 mM), prehydrolyzed at room temperature for 5 min, and then AA was added into the mixture to initiate the reduction of HAuCl_4_ and the growth of Au particles. The concentrations of HAuCl_4_ and AA were fixed at 0.25 and 1.5 mM unless stated especially, and that of APTES was changed in a range of 0–2.5 mM, corresponding to the molar ratio of HAuCl_4_ and APTES (*R*_Au/APTES_), in the range from 1:0 to 1:10. Figure 2 shows UV–vis spectra of the Au particles prepared with different *R*_Au/APTES_ values. In the absence of APTES (*R*_Au/APTES_ of 1:0), the resulting Au hydrosol was red in color, corresponding to an LSPR peak with λ_max_ centered at 520 nm, suggesting the formation of sphere-like Au particles. When the value of *R*_Au/APTES_ was elevated to 1:2, 1:4, 1:6, 1:8, and 1:10, the resulting hydrosols became purple, light blue, blue, bluish green, and bluish gray in color, and correspondingly, λ_max_ of the LSPR peak shifted to 529, 653, 636, 677, and 685 nm, respectively, suggesting the increased size and/or more anisotropic character of the resulting Au particles. Transmission electron microscopy (TEM) observations and the histogram of the numerical size distributions showed that the Au particles grown at *R*_Au/APTES_ of 1:0 were sphere-like in shape, with an average size of 10 ± 1.4 nm (Figure 3a). When the *R*_Au/APTES_ was 1:2, the resulting Au particles became less regular in shape, with an average size of 14 ± 1.9 nm (Figure 3b). Such a slight increase in the size of the Au particles (from 10 ± 1.4 to 14 ± 1.9 nm) indicated that the red shift in λ_max_ of the LSPR peak (from 520 to 529 nm) and the change in color (from red to purple) of the Au hydrosols are primarily attributed to the change in the morphology of the Au particles. When the value of *R*_Au/APTES_ was elevated to 1:4, the resulting Au particles became flower-like in shape, with an average size of 73 ± 4.1 nm (Figure 3c). Further increasing the *R*_Au/APTES_ to 1:6, 1:8, and 1:10 resulted in the formation of the Au NFs with longer branches with almost the same average size of 49 ± 2.3 nm (Figure 3d–f). It was noted that the Au particles prepared at an *R*_Au/APTES_ value of 1:4 presented a much red-shifted λ_max_ of the LSPR peak than those prepared at an *R*_Au/APTES_ value of 1:6 (653 vs 636 nm). It was likely that such a red shift in λ_max_ of the LSPR peak is related to the difference in the size of the Au NFs (73 ± 4.1 vs 49 ± 2.3 nm) since there was no superiority in the branch length for the Au NFs prepared at an *R*_Au/APTES_ value of 1:4 compared with that of the Au NFs prepared at an *R*_Au/APTES_ value of 1:6. Based on these results, it was deduced that the increase in the *R*_Au/APTES_ value facilitates the formation of Au NFs with an increased branch length and thus red-shifted λ_max_ of the LSPR peak. A control experiment was carried out to further elucidate the effect of APTES on growth of the Au NFs. When fresh APTES was used instead of the prehydrolyzed APTES in the reaction, the branch length of the Au NFs, formed at *R*_Au/APTES_ 1:8, became shorter, accompanied by a blue shift in λ_max_ of the LSPR peak (677 vs 612 nm, Figure S2), indicating that the prehydrolyzed APTES is more effective in promoting the formation of Au NFs than the fresh one.

**Figure 4:**
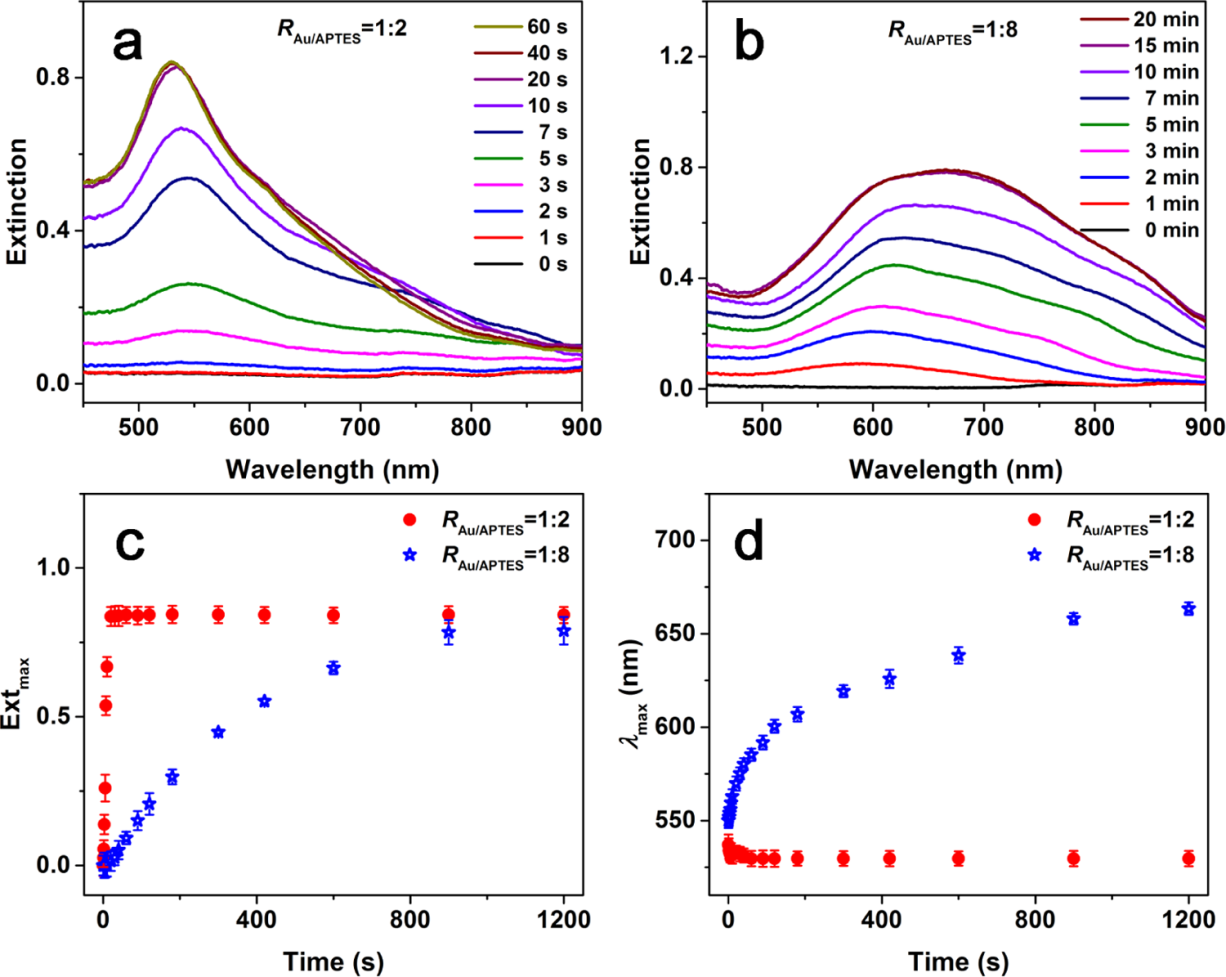
Temporal evolution in UV–vis spectra of the reactions carried out at *R*_Au/APTES_ of (a) 1:2 and (b) 1:8. Summarized temporal evolution in (c) maximum extinction and (d) λ_max_ of the LSPR peak of the reaction solutions carried out at *R*_Au/APTES_ of 1:2 (red) and 1:8 (blue).

**Figure 5:**
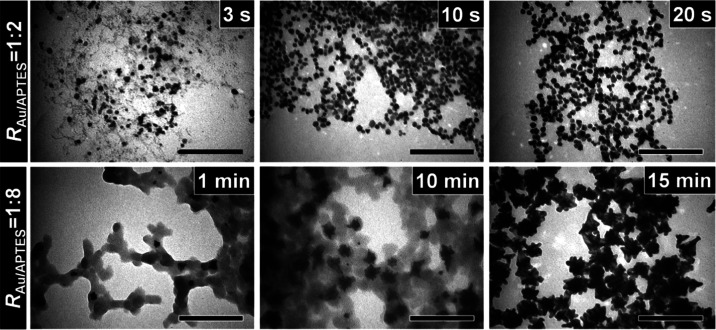
Temporal evolution in size/shape of the Au particles grown in the reactions carried out at *R*_Au/APTES_ of (top panel) 1:2 and (lower panel) 1:8. The scale bars in all of the TEM images are 200 nm.

Temporal evolutions in UV–vis absorption spectra of the reactions carried out at two typical *R*_Au/APTES_ values, i.e., 1:2 and 1:8, were followed to further understand the effect of APTES on the morphology of the resulting Au particles. In the reaction carried out at *R*_Au/APTES_ of 1:2, an LSPR peak with a λ_max_ at 535 nm became observable after 3 s of the reaction, whose intensity increased rapidly and remained almost unchanged after 20 s of the reaction (Figure 4a,c), accompanied by a slight blue shift in λ_max_ of the LSPR peak from 535 to 529 nm (Figure 4d). For the reaction carried out at *R*_Au/APTES_ of 1:8, an LSPR peak with λ_max_ at 580 nm became observable after 1 min of the reaction, whose intensity increased gradually and then remained almost unchanged after 15 min of the reaction (Figure 4b,c), accompanied by an obvious red shift in λ_max_ of the LSPR peak from 580 to 677 nm (Figure 4d), indicating the reduced reactivity of the gold precursor and the facilitated growth of the Au NFs under the elevated APTES concentration. TEM observations showed that in the reaction with *R*_Au/APTES_ of 1:2, Au particles with an average size of ca. 10 nm were observable after 3 s of the reaction. When the reaction was prolonged to 10 and 20 s, the average size of the Au particles only increased slightly to ca. 13 and 14 nm (Figure 5, top panel). Taking the UV–vis spectral results and the TEM observations together, it was deduced that there is continuous nucleation in the reaction carried out at this low *R*_Au/APTES_ value (1:2), attributed to high activity of the gold precursor, contributing to the formation of small sphere-like Au particles (Figure S3a). In the reaction solution with *R*_Au/APTES_ of 1:8, the Au particles formed after 1 min of the reaction were irregular in shape, which evolved into a more flower-like morphology after 15 min of the reaction (Figure 5, lower panel, and Figure S3b). These results suggested the promoted formation of Au NFs at a high *R*_Au/APTES_ value (1:8), possibly due to a decrease in the activity of the gold precursor and thus slowed down the growth of the Au particles at a high APTES concentration.^7^

**Figure 6:**
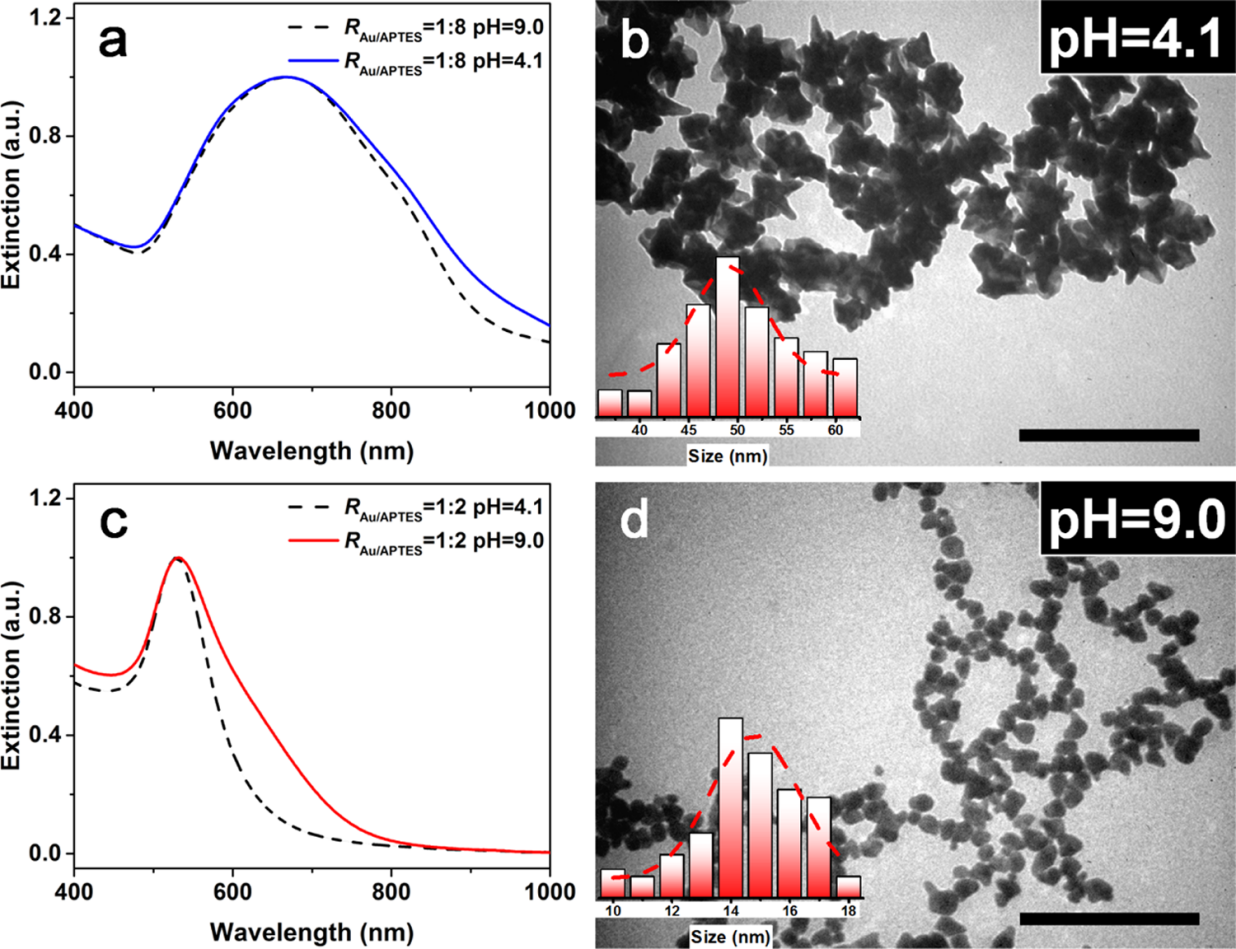
(a) Normalized UV–vis spectrum and (b) TEM image of the Au particles grown in the reaction carried out at *R*_Au/APTES_ of 1:8 and pH 4.1. Normalized UV–vis spectrum of the Au NFs grown at the same *R*_Au/APTES_ value and pH 9.0 was given as a dotted line for comparison. (c) Normalized UV–vis spectrum and (d) TEM image of the Au particles grown in the reaction carried out at *R*_Au/APTES_ of 1:2 and pH 9.0. Normalized UV–vis spectrum of the Au particles grown at the same *R*_Au/APTES_ value and pH 4.1 was given as a dotted line for comparison. Insets show histograms of the numerical size distribution of the particles. The scale bars in all of the TEM images are 200 nm.

It was expected that the change in *R*_Au/APTES_ may affect pH of the reaction solution and thus reactivity of the Au precursor since APTES is a weak base in character. When the value of *R*_Au/APTES_ was set to be 1:2, initial pH of the reaction solution was measured to be 4.1, which increased to 9.0 when the value of *R*_Au/APTES_ was elevated to 1:8. To elucidate the effect of pH on growth of the Au particles, initial pH of the reaction solution with *R*_Au/APTES_ of 1:8 was adjusted from 9.0 to 4.1 by addition of HNO_3_, and that of the reaction solution with *R*_Au/APTES_ of 1:8 was adjusted from 4.1 to 9.0 by addition of LiOH. For the reactions carried out at *R*_Au/APTES_ of 1:8, λ_max_ of the LSPR peak remained almost unchanged (Figure 6a) and the resulting Au particles were still flower-like in shape (Figure 6b) after pH of the reaction solution was lowered to 4.1. For the reactions carried out at *R*_Au/APTES_ of 1:2, λ_max_ of the LSPR peak remained almost unchanged (Figure 6c) and the resulting Au particles were still primarily sphere-like in shape (Figure 6d) after pH of the reaction solution was elevated to 9.0. It was noted that extinction in the 600–800 nm window was almost unchanged when pH of the reaction solution with *R*_Au/APTES_ of 1:8 was lowered from 9.0 to 4.1, and that increased to some extent when pH of the reaction solution with *R*_Au/APTES_ of 1:2 was elevated from 4.1 to 9.0 (Figure 6a,c), indicating the slightly promoted anisotropic character of the resulting Au particles, possibly due to the reduced activity of the gold precursor at elevated pH.^35,36^ These results implied that the ratio of *R*_Au/APTES_ is more effective than pH in tuning shape of the resulting Au particles since the change in pH was not enough to induce the change in the shape of the resulting Au particles at either a low (1:2) or high (1:8) *R*_Au/APTES_ ratio.

**Figure 7:**
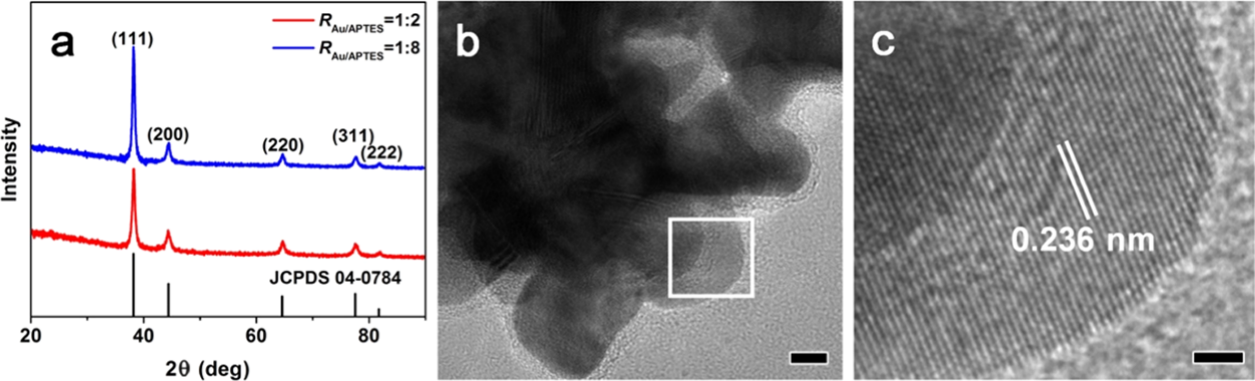
(a) XRD patterns of the Au particles prepared at *R*_Au/APTES_ of 1:2 and 1:8. The standard XRD pattern of fcc gold (JCPDS 04-0784) is also given for comparison. (b) Enlarged TEM image of the Au NFs prepared at *R*_Au/APTES_ of 1:8 and (c) HRTEM image of the labeled branch in (b). The scale bars in (b, c) are 5 and 2 nm, respectively.

X-ray diffraction (XRD) patterns (Figure 7a) were collected to further understand the effect of APTES on growth of the Au particles. Both the Au particles prepared at *R*_Au/APTES_ of 1:2 and 1:8 presented diffraction peaks at 38.2, 44.4, 64.8, 77.9, and 81.7°, corresponding to the (111), (200), (220), (311), and (222) planes, well matched with those of the fcc gold. It was noted that the intensity ratio of the (111) and (200) planes (*I*_111_/*I*_200_) was 3.44 for the Au particles prepared at *R*_Au/APTES_ of 1:2, which increased to 4.35 for those prepared at *R*_Au/APTES_ of 1:8, indicating the preferential growth of the Au particles along the (111) facet under the elevated APTES concentration, contributing to formation of the anisotropic Au NFs.^17^ High-resolution transmission electron microscopy (HRTEM) observations revealed that branches of the Au NFs were mainly composed of a lattice with a fringe spacing of 0.236 nm (Figure 7b,c), corresponding to the (111) plane of fcc gold, consistent with the XRD results. Thus, it was concluded that the increased concentration of APTES is favorable for preferential growth of Au particles along the (111) plane of fcc gold, resulting in the formation of Au NFs.

**Figure 8:**
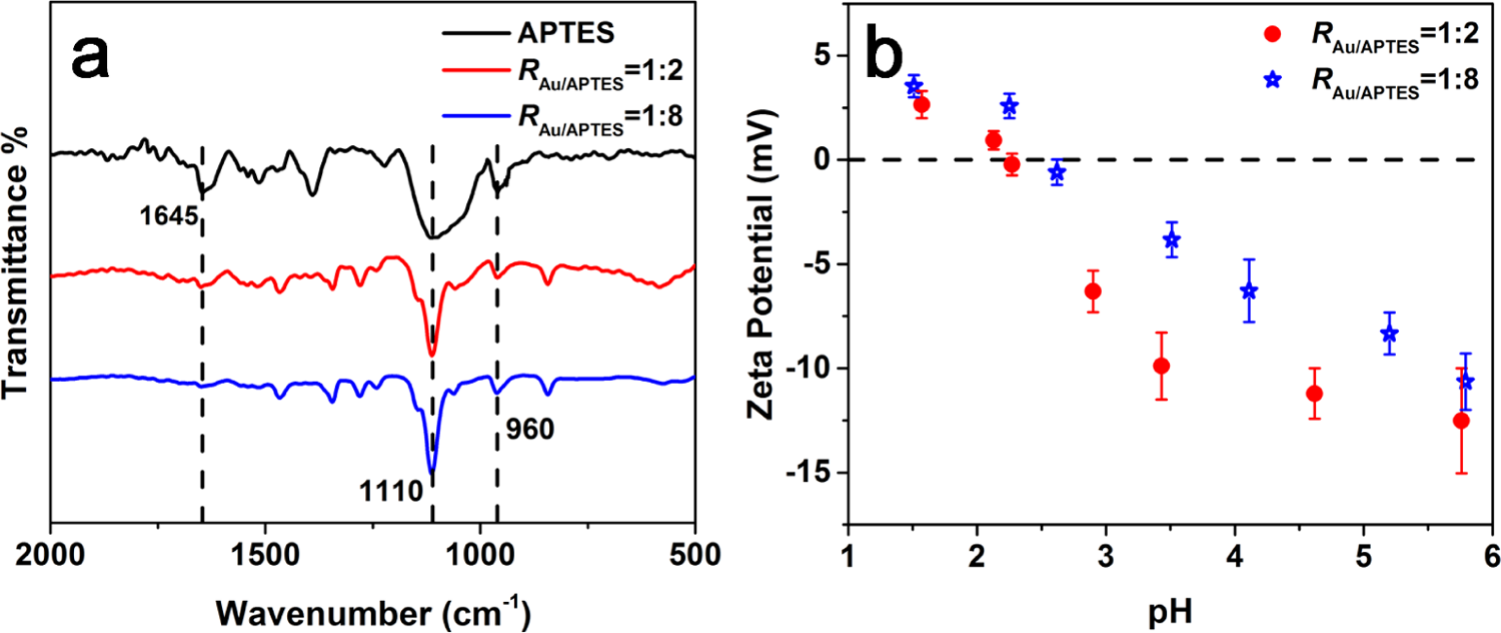
(a) FTIR spectra of APTES and the Au particles prepared at *R*_Au/APTES_ of 1:2 and 1:8. (b) Variations in ζ-potential of the Au particles prepared at *R*_Au/APTES_ of 1:2 and 1:8 with pH.

**Figure 9:**
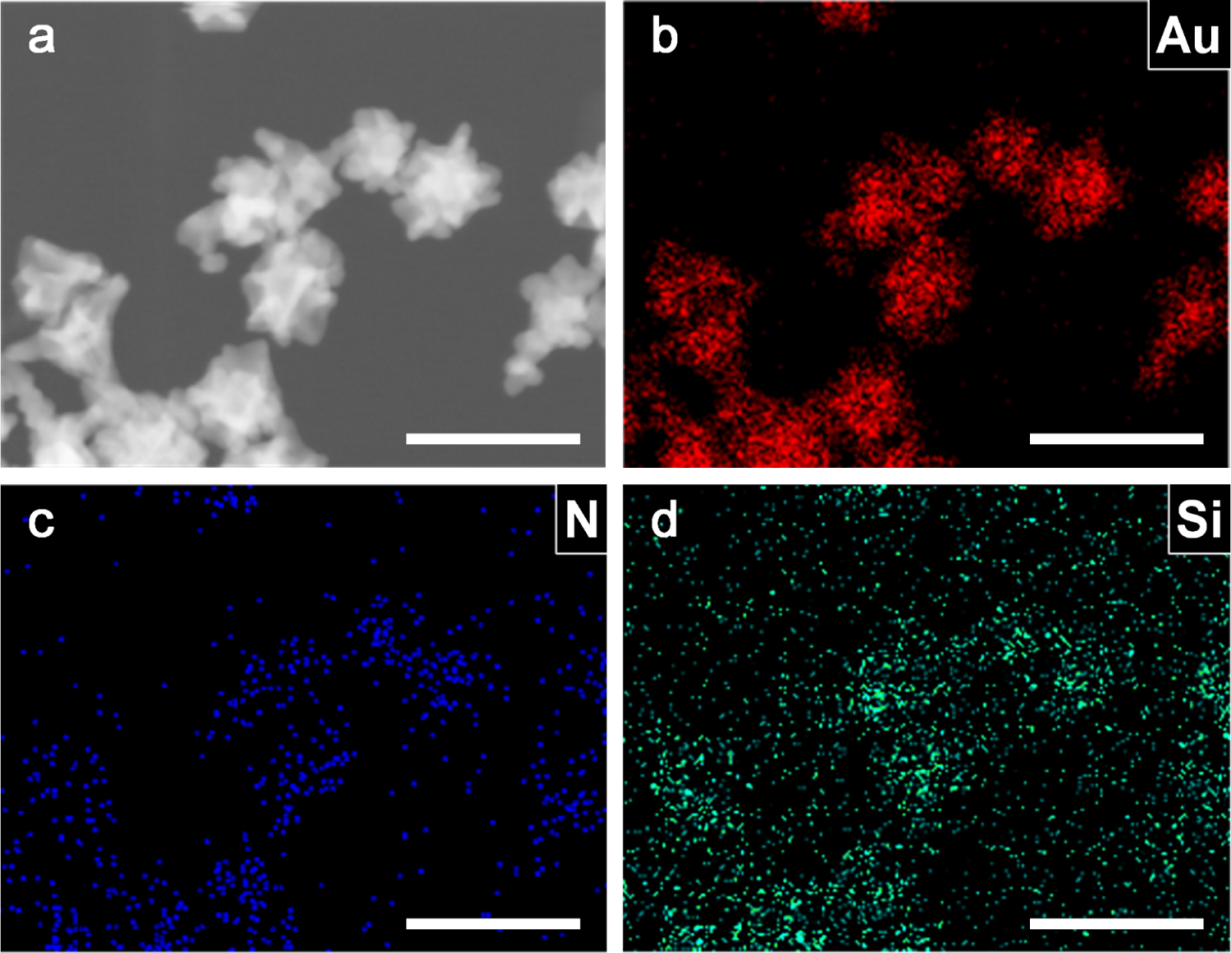
(a) STEM image of the Au NFs prepared at *R*_Au/APTES_ at 1:8 and the corresponding element mapping images of (b) Au, (c) N, and (d) Si. The scale bars in all of the images are 100 nm.

The presence of APTES on the surface of the Au particles was revealed by Fourier transform infrared (FTIR) spectra, ζ-potential, and energy dispersive X-ray spectroscopy (EDS) measurements. The stretching vibrations of Si–OH at 960 cm^–1^ and Si–O–Si at 1110 cm^–1^,^30,37^ attributed to the condensed and/or hydrolyzed APTES, were identifiable in the Au particles prepared at either a low (1:2) or high (1:8) *R*_Au/APTES_ ratio, suggesting the existence of APTES on the particle surfaces. It was noted that the N–H bending vibration of APTES at 1645 cm^–1^ became weaker in the presence of the Au particles (Figure 8a), indicating that APTES is anchored onto the particle surface via its amino group.^38^ The flower-like Au particles, prepared at an *R*_Au/APTES_ ratio of 1:8, presented an isoelectric point around 2.8, similar to that (ca. 2.5) of the sphere-like Au particles prepared at the *R*_Au/APTES_ ratio of 1:2 (Figure 8b), indicating the similar surface character of the flower-like and sphere-like Au particles.^39^ Scanning transmission electron microscopy (STEM) and the corresponding EDS elemental mapping images revealed the coexistence of Au, N, and Si elements on the surface of the Au NFs (Figure 9), further proving the existence of APTES on the Au particle surface.

The stability of the Au NFs was evaluated by dynamic light scattering and UV–vis spectra. After being kept at room temperature for 60 h, there were almost no changes in hydrodynamic diameters and polydispersity, as well as UV–vis spectra, of the Au NFs prepared at *R*_Au/APTES_ of 1:8 (Figure S4), indicating excellent stability of the Au NFs, attributed to the protection effect of the amino-containing organosilane (APTES) toward the Au NFs. To evaluate the reproducibility of our method, five batches of the Au NFs were prepared at *R*_Au/APTES_ of 1:8. The Au NFs, prepared from the five different batches, presented very similar hydrodynamic diameters and an LSPR peak with almost the same λ_max_ (Figure S5), indicating the good reproducibility of the APTES-directed approach for the synthesis of Au NFs.

**Figure 10:**
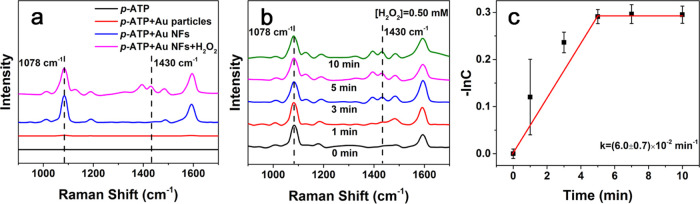
(a) SERS spectra of 1.0 μM *p*-ATP in the presence of the Au particles prepared at *R*_Au/APTES_ of 1:2 (red) and the Au NFs prepared at *R*_Au/APTES_ of 1:8 in the absence (blue) and the presence (purple) of H_2_O_2_. The Raman spectrum of the 0.2 mM *p*-ATP solution (black) is also given for comparison. (b) Normalized time-dependent SERS spectra of 1.0 μM *p*-ATP in the presence of the Au NFs prepared at *R*_Au/APTES_ of 1:8 and 0.50 mM H_2_O_2_. (c) Corresponding fitting curve of the negative natural logarithm of the estimated relative concentration of *p*-ATP vs the reaction time derived from (b).

Dimerization of *p*-aminothiophenol (*p*-ATP) into dimercaptoazobenzene (DMAB), a reaction with great significance in the fields of pharmaceuticals, dyes, food additives, etc.,^40^ was employed as a model reaction to evaluate catalytic and SERS performances of the as-prepared Au particles. The solution of *p*-ATP (0.2 mM) presented very weak Raman signals at 1078 and 1588 cm^–1^, assigned to the stretching vibrations of C–S and C–C of *p*-ATP, respectively.^41^ In the presence of the sphere-like Au particles, prepared at *R*_Au/APTES_ of 1:2, the signals were only enhanced slightly, corresponding to an enhanced factor (EF) of 1 × 10^3^. In the presence of the Au NFs prepared at *R*_Au/APTES_ of 1:8, the signals were enhanced greatly, corresponding to an enhanced factor (EF) of 2 × 10^5^, comparable to performance of most of the anisotropic Au particles in the literature (Table S1).^3,13−15,42−44^ After the addition of H_2_O_2_, a new Raman band at 1430 cm^–1^, assigned to ring-stretching vibrations associated with the N=N moiety, became observable (Figure 10a), indicating that the Au NFs are capable of catalyzing the dimerization of *p*-ATP into DMAB in the presence of H_2_O_2_, via conversion of the amino groups in *p*-ATP into N=N bond induced by ^•^OH generated from the decomposition of H_2_O_2_.^41,45,46^ In the time-dependent SERS spectra, which were normalized according to the signal of the C–S bond at 1078 cm^–1^, the intensity of the signal at 1430 cm^–1^, assigned to the N=N bond, increased gradually with the reaction time (Figure 10b), indicating the gradual conversion of *p*-ATP into DMAB with the proceeded reaction. The intensity ratio of the Raman signals at 1430 and 1078 cm^–1^, which may represent the amount of DMAB generated from the dimerization of *p*-ATP,^47,48^ increased with the reaction time and reached a platform within 5 min. The results indicated that the dimerization of *p*-ATP can be monitored in situ using the Au NFs as a SERS substrate. Figure 10c further shows that the negative natural logarithm of the concentration of *p*-ATP, estimated from the intensity ratio at 1430 and 1078 cm^–1^, increases linearly with time at the early stage of the reaction, suggesting that the dimerization of *p*-ATP with the catalysis of the Au NFs is a first-order reaction^46,49^ with a rate constant of (6.0 ± 0.7) × 10^–2^ min^–1^.

## Conclusions

In summary, in this work, we demonstrated a facile approach for the synthesis of Au NFs using APTES as a shape-directing agent. With increasing the molar ratios of HAuCl_4_ to APTES, the morphology of the Au particles evolved from sphere-like to flower-like, accompanied by a red shift in λ_max_ of the LSPR peak of the Au particles from 520 to 685 nm. In this approach, an increase in the concentration of APTES promotes the preferential growth of Au particles along the (111) facet, and thus the formation of Au NFs, more anisotropic in character. The as-prepared Au NFs were qualified for simultaneously catalyzing the dimerization of *p*-ATP and monitoring the reaction via in situ SERS detection. It is expected that such an APTES-directed approach would highly benefit the development of Au NF-based chemical catalysis and SERS detection.

## Experimental Section

### Materials

Chlorauric acid tetrahydrate (HAuCl_4_·4H_2_O, 99%) was purchased from Sinopharm Chemical Reagent Co., Ltd. 3-Aminopropyltriethoxysilane (APTES, ≥98%) was purchased from Sigma-Aldrich. Ascorbic acid (AA, ≥99%) was purchased from Alfa Aesar and *p*-aminothiophenol (*p*-ATP, 97%) was purchased from Aladdin Industrial Incorporated. Hydrogen peroxide (H_2_O_2_) and nitric acid (HNO_3_) were purchased from Beijing Chemical Work and lithium hydroxide (LiOH) was purchased from Tianjin Chemical Co. All reagents were analytical in grade and used without further purification. High-purity water (Pall Purelab Plus) with a resistivity of 18.2 MΩ·cm was used in all experiments. All glassware used was cleaned in a bath of freshly prepared aqua regia solution (HCl/HNO_3_, 3:1) and a chromic acid lotion, and then rinsed thoroughly with high-purity water before use. All of the experiments were carried out at room temperature (25 ± 2 °C) unless stated especially.

### Characterization

UV–vis spectra were recorded on a Shimadzu UV-1800 UV–vis spectrophotometer. Temporal evolutions in UV–vis spectra were acquired using an Ocean Optics HR4000CG-UV–NIR high-resolution spectrophotometer. TEM images were taken on a JEOL JEM-2010 electron microscope with an acceleration voltage of 100 kV. HRTEM images, STEM images, and EDS measurements were taken on a JEOL JEM-2100F scanning transmission electron microscope with an acceleration voltage of 200 kV, equipped with a high-angle annular dark-field (HAADF) detector. The dispersions of Au particles were dropped onto carbon-coated copper grids and then dried in air before the TEM and HRTEM observations. Mass spectrometric analysis was performed on a Thermo Fisher Orbitrap Fusion Tribrid mass spectrometer with negative-ion detection modes. Hydrodynamic diameters and ζ-potentials were measured on a Brookhaven ZetaPALS apparatus. FTIR spectra were recorded using a Thermo Scientific Nicolet iS50 FTIR spectrophotometer in a scanning range from 500 to 2000 cm^–1^ with 64 scans at a resolution of 8 cm^–1^. The Au particles were collected by centrifugation and then dried in vacuum and mixed with KBr powders before FTIR measurements. XRD patterns were collected on a PANalytical Empyrean X-ray diffractometer equipped with a graphite monochromator (Cu Kα radiation, λ = 1.54 Å) at a scanning speed of 5° min^–1^. Raman and SERS spectra were acquired on a Horiba LabRAM HR Evolution spectrophotometer equipped with a 785 nm laser in a quartz cell with an optical path of 1 cm. All of the Raman measurements were performed in the solution phase with an accumulation time of 60 s at a laser power of 50 mW, which almost showed no photothermal effect on the dispersion of the Au NFs (Figure S6).

### Preparation of Au Particles

APTES was added to high-purity water to form the 3 mM aqueous solution and kept at room temperature for 5 min. After that, 25 μL of an aqueous solution of HAuCl_4_ (100 mM) and different volumes of the APTES aqueous solution (3 mM) were mixed under magnetic stirring (300 rpm) at room temperature with the final volume fixed at 10 mL, followed by the addition of 150 μL of an aqueous solution of AA (100 mM). The concentrations of HAuCl_4_ and AA were set at 0.25 and 1.5 mM and that of APTES varied from 0 to 0.5, 1.0, 1.5, 2.0, and 2.5 mM in the final solutions, corresponding to the molar ratios of HAuCl_4_ and APTES (*R*_Au/APTES_) of 1:0, 1:2, 1:4, 1:6, 1:8, and 1:10, respectively. After 30 min, the resulting Au particles were collected by centrifugation (3000 rpm, 10 min) and then redispersed in pure water for further characterization.

### Catalyzing and In Situ SERS Monitoring the Dimerization of *p*-ATP

Overall, 10 μL of 0.2 mM *p*-ATP was added in 2 mL of a dispersion of Au particles (0.02 nM) prepared at *R*_Au/APTES_ 1:2 and 1:8, respectively, and then incubated for 10 min at room temperature. After transferring the abovementioned mixture into 1 cm quartz cuvettes, 0.50 mM H_2_O_2_ was added into the mixture, followed by SERS measurements immediately.

The Raman enhancement factor (EF) was calculated using the following equation^28^
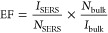
1where *I*_SERS_ and *I*_bulk_ are Raman intensities with and without Au particles and *N*_SERS_ and *N*_bulk_ are the number of probe molecules adsorbed on Au particles and in the bulk solution sample.

## Supporting Information Available

Negative differentiation curve of the conductivity of the aqueous solution of APTES (Figure S1), normalized UV–vis spectrum and the TEM image of the Au particles prepared using fresh APTES (Figure S2), enlarged TEM images of the Au particles prepared at *R*_Au/APTES_ of 1:2 and 1:8 (Figure S3), temporal evolution in hydrodynamic diameters and polydispersity of the Au NFs (Figure S4), variations in the hydrodynamic diameter and polydispersity of the Au NFs prepared from the five different batches (Figure S5), temperature change of dispersion of the Au NFs with the laser irradiation time (Figure S6), and brief summary of the SERS performance of anisotropic Au particles prepared in our work and those reported in the literature (Table S1) (PDF)
