# Supplementary material for: Au Nanoflowers for Catalyzing and In Situ Surface-Enhanced Raman Spectroscopy Monitoring of the Dimerization of p-Aminothiophenol
Source: ACS Omega. 2021 Sep 21;6(39):25720–8. doi: 10.1021/acsomega.1c03933 (PMC8495860; doi:10.1021/acsomega.1c03933)
Supplement: Supplementary file 1 — ao1c03933_si_001.pdf [file ao1c03933_si_001.pdf]

Supporting Information

Au Nanoflowers for Catalyzing and In Situ Surface-Enhanced Raman Spectroscopy Monitoring of the Dimerization of *p*-Aminothiophenol

Jingwen Ba[†], *Yandong Han*[‡], *Xiaoyu Zhang*[†], *Lijuan Zhang*[†], *Shuhan Hui*[†], *Zhenzhen Huang*[*,†] *and Wensheng Yang*[*,†]

[†]State Key Laboratory of Inorganic Synthesis and Preparative Chemistry, College of Chemistry, Jilin University, Changchun 130012, China.

[‡]Institute of Molecular Plus, Tianjin University, Tianjin 300072, China.

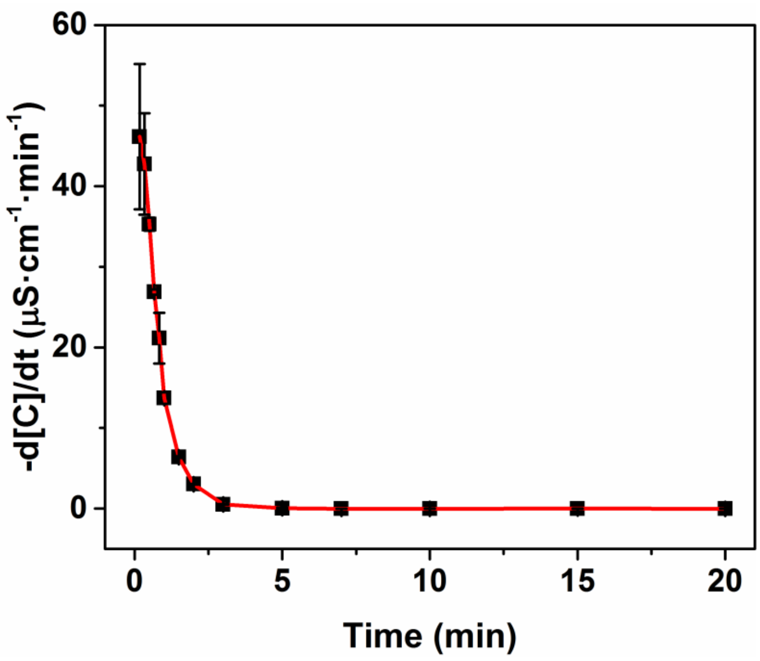

Figure S1. *Negative differentiation curve of the conductivity of the aqueous solution of APTES (3 mM). A -d[C]/dt value of 46 μS·cm^{-1}·min^{-1} was observed after 10 s of the reaction. Absence of the maximum -d[C]/dt value in the negative differentiation curve, which presents approximately the maximum condensation rate of the organosilane agent (APTES), meaning that the maximum condensation rate of APTES is ≥ 46 μS·cm^{-1}·min^{-1} in the aqueous solution (see Ref. 33 in the text).*

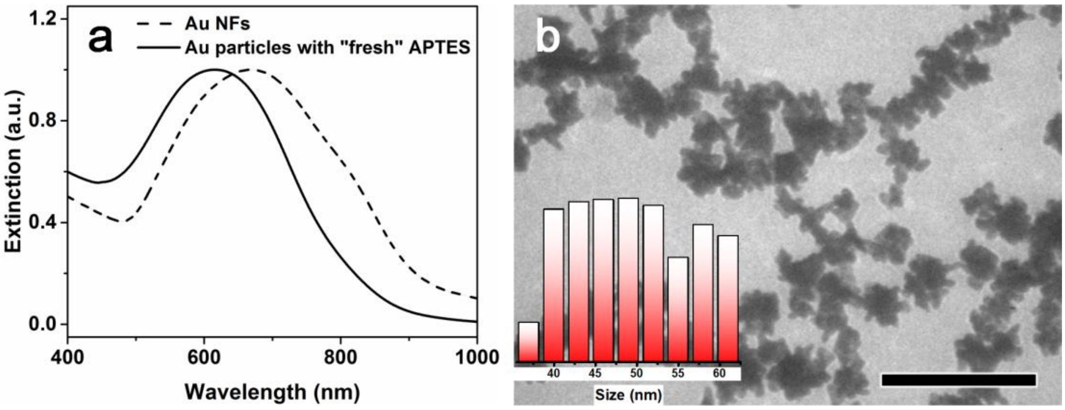

Figure S2. *(a) Normalized UV−vis spectrum (solid line) and (b) TEM image of the Au particles prepared by using fresh APTES at $R_{Au/APTES}$ of 1:8. Normalized UV−vis spectrum of the Au NFs prepared by using the APTES prehydrolyzed for 5 min at the same $R_{Au/APTES}$ value was given as a dotted line for comparison. The blue-shifted λ_{max} of the LSPR peak (677 vs. 612 nm), as well as the shorter branch length of the resulting Au NFs prepared with the fresh APTES (see TEM image in Figure 3e for comparison) indicated that the prehydrolyzed APTES is more effective in promoting the formation of Au NFs than the fresh one.*

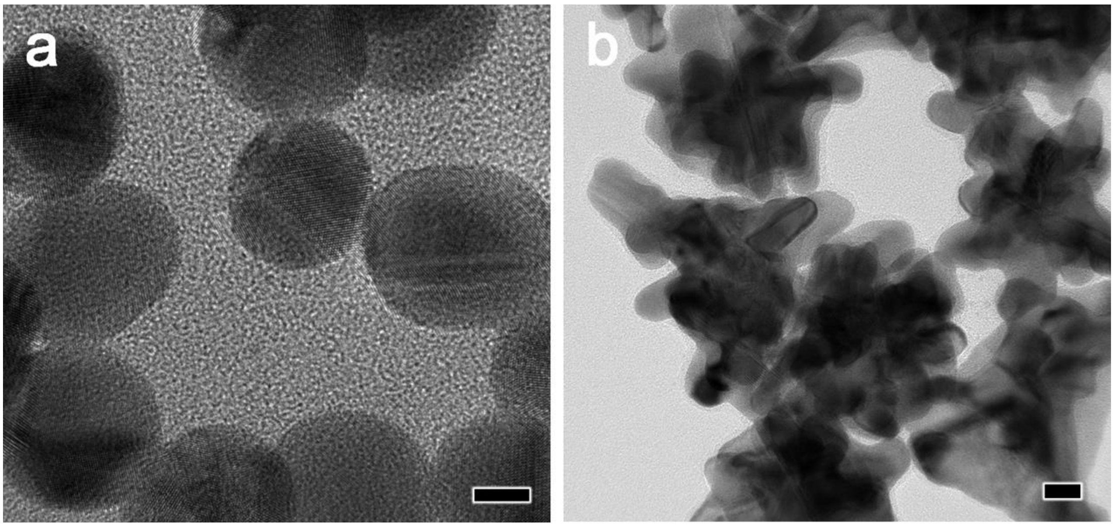

Figure S3. *Enlarged TEM images of the Au particles prepared at* $R_{Au/APTES}$ *of (a) 1:2 and (b) 1:8. The scale bares are 5 nm and 10 nm, respectively. The Au particles prepared at* $R_{Au/APTES}$ *of 1:2 were primarily sphere-like in shape and these at* $R_{Au/APTES}$ *of 1:8 were typical flower-like in shape.*

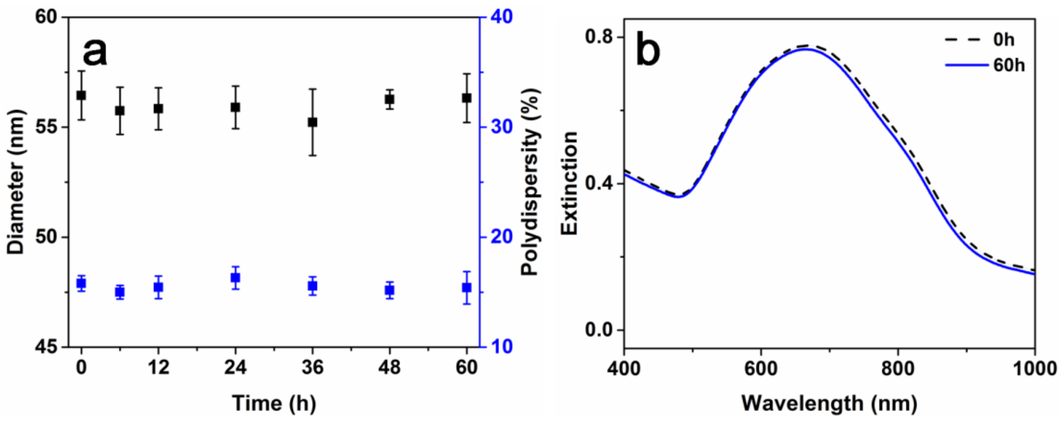

Figure S4. *(a) Temporal evolution in hydrodynamic diameters (black) and polydispersity (blue) of the Au NFs prepared at $R_{Au/APTES}$ of 1:8. (b) UV–vis spectra of the Au NFs prepared at $R_{Au/APTES}$ of 1:8 taken immediately and after being kept at room temperature for 60 h. The almost unchanged hydrodynamic diameters and polydispersity, as well as the very similar UV–vis spectra indicated the good stability of the Au NFs, possibly due to the protection effect of APTES anchored on their surface (see Figure 8 in the text).*

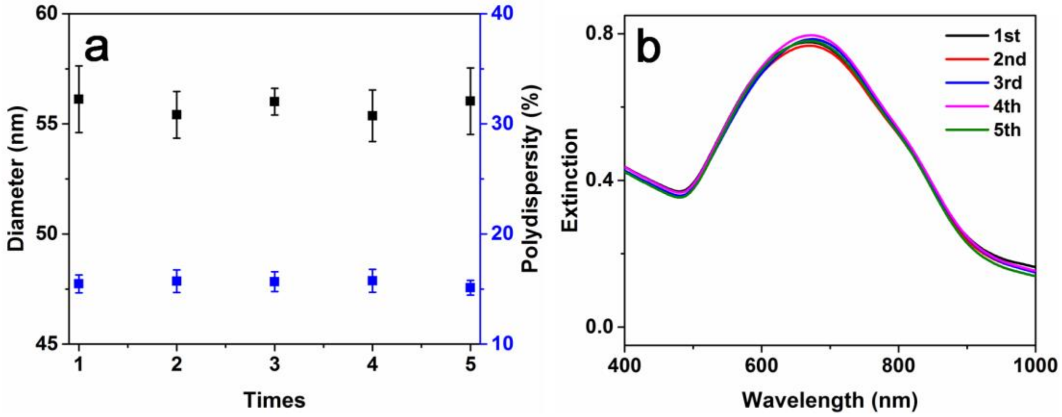

Figure S5. *(a) Variations in hydrodynamic diameter (black) and polydispersity (blue dots) of the Au NFs prepared from the five different batches carried out at* $R_{Au/APTES}$ *of 1:8. (b) UV–vis spectra of the Au NFs prepared from the five different batches. The very similar hydrodynamics diameter and polydispersity, as well as the almost same spectra, indicated the good reproducibility of this APTES-directed approach for the synthesis of Au NFs.*

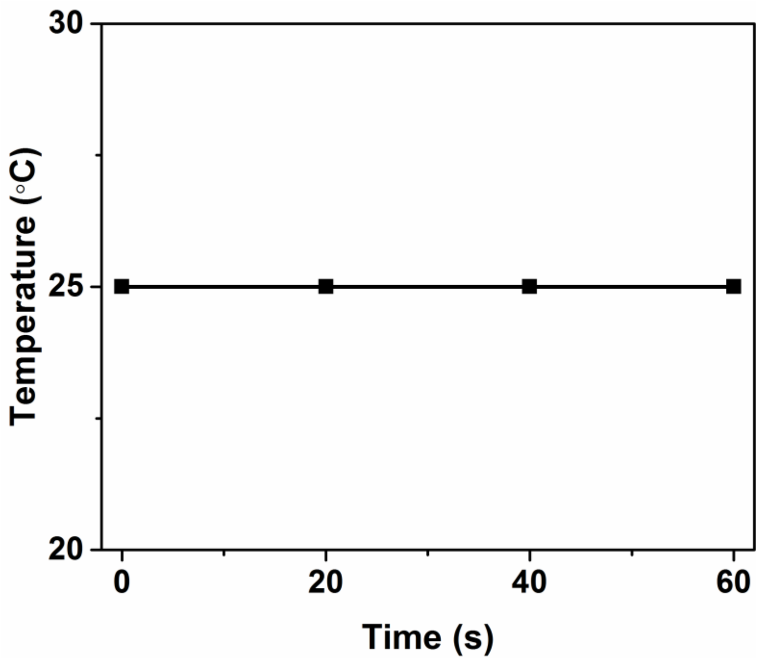

Figure S6. *Temperature change of dispersion of the Au NFs prepared at* $R_{Au/APTES}$ *of 1:8 with the laser irradiation time (785 nm, 50 mW). No obvious change in temperature was observed, meaning the negligible photothermal effect of the Au NFs under the irradiation.*

Table S1. *Brief summary of the SERS performance of anisotropic Au particles prepared in our work and these reported in literature*

Morphology	SERS remarks	SERS *EF*	Ref. in the text
hollow-channel gold nanoflowers	biphenyl-4-thiol	10^5	(3)
gold nanostars	4-nitrothiophenol	9.5×10^3–1.5×10^4	(13)
gold nanoflowers	4-mercaptobenzoic	1.40×10^5	(14)
spinous-like Au nanostructures	crystal violet	10^5–10^7	(15)
gold nanoflower chains	*p*-aminothiophenol	2.9×10^5	(42)
hollow porous gold nanoshells	rhodamine 6G	~3.0×10^5	(43)
capsid-like gold	4-mercaptophenol	1.7×10^5	(44)
**Au nanoflowers**	***p*-aminothiophenol**	**2×10^5**	**this work**
